# Cannabidiol and cannabis-inspired terpene blends have acute prosocial effects in the BTBR mouse model of autism spectrum disorder

**DOI:** 10.3389/fnins.2023.1185737

**Published:** 2023-06-16

**Authors:** Jenika Staben, Megan Koch, Keelee Reid, Jessica Muckerheide, Lauren Gilman, Finn McGuinness, Sarina Kiesser, Iain W. H. Oswald, Kevin A. Koby, Thomas J. Martin, Joshua S. Kaplan

**Affiliations:** ^1^Department of Psychology, Behavioral Neuroscience Program, Western Washington University, Bellingham, WA, United States; ^2^Scientific Technical Services, Western Washington University, Bellingham, WA, United States; ^3^Department of Research and Development, Abstrax Tech, Inc., Tustin, CA, United States

**Keywords:** cannabidiol, CBD, cannabinoid, cannabis, autism spectrum disorder, social behavior, terpenes, volatile organic compounds

## Abstract

**Introduction:**

Cannabidiol (CBD) is a non-intoxicating phytocannabinoid with increasing popularity due to its purported therapeutic efficacy for numerous off-label conditions including anxiety and autism spectrum disorder (ASD). Those with ASD are commonly deficient in endogenous cannabinoid signaling and GABAergic tone. CBD has a complex pharmacodynamic profile that includes enhancing GABA and endocannabinoid signaling. Thus, there is mechanistic justification for investigating CBD’s potential to improve social interaction and related symptoms in ASD. Recent clinical trials in children with ASD support CBD’s beneficial effects in numerous comorbid symptoms, but its impact on social behavior is understudied.

**Methods:**

Here, we tested the prosocial and general anxiolytic efficacy of a commercially available CBD-rich broad spectrum hemp oil delivered by repeated puff vaporization and consumed via passive inhalation in the female cohort of the BTBR strain, a common inbred mouse line for preclinical assessment of ASD-like behaviors.

**Results:**

We observed that CBD enhanced prosocial behaviors using the 3-Chamber Test with a different vapor dose-response relationship between prosocial behavior and anxiety-related behavior on the elevated plus maze. We also identified that inhalation of a vaporized terpene blend from the popular OG Kush cannabis strain increased prosocial behavior independently of CBD and acted together with CBD to promote a robust prosocial effect. We observed similar prosocial effects with two additional cannabis terpene blends from the Do-Si-Dos and Blue Dream strains, and further reveal that these prosocial benefits rely on the combination of multiple terpenes that comprise the blends.

**Discussion:**

Our results illustrate the added benefit of cannabis terpene blends for CBD-based treatment of ASD.

## Introduction

*Cannabis sativa* L synthesizes hundreds of distinct chemicals (ElSohly and Slade, 2005). Differences in this composition across genetic strains and products confers unique psychopharmacological effects and impacts the purported therapeutic effects (Sholler et al., 2020). Although cannabis has been used medicinally for millennia (Russo, 2007), the individual chemical or combination of chemicals responsible for symptomatic relief across numerous clinical indications are just starting to be understood. Δ^9^-tetrahydrocannabinol (Δ^9^-THC), which is responsible for the euphoric and intoxicating nature of cannabis, has historically drawn the bulk of research attention (Liu et al., 2020). However, the shared and complementary pharmacodynamic mechanisms across numerous phytocannabinoids has stoked research interest into the impact that non-intoxicating phytocannabinoids, like cannabidiol (CBD), may have across different therapeutic domains (Russo, 2011, 2019; Mandolino et al., 2019). Furthermore, volatile organic compounds, although not unique to *Cannabis sativa* L, are synthesized by the plant in unique “blends” and confer it’s unique odor and flavor (Sommano et al., 2020). Terpenes are a category of volatile organic compounds that are abundantly produced and may interact with the phytocannabinoids themselves or act as cannabimimetics to confer their own therapeutic properties (LaVigne et al., 2021). Together, this vast diversity of phytocannabinoids and cannabis terpene blends exposes the immense complexity of the pharmacodynamic interactions in whole-plant cannabis extracts that may impact its medicinal characteristics. It also reveals optimization potential for developing cannabis-based medicines with improved efficacy or extended effective dose ranges (Ferber et al., 2020).

Cannabis use has increased along with legalized access to medicinal and recreational products (Han et al., 2018; Lapham et al., 2022). Currently, the only approved cannabis-derived medicine by the United States Food and Drug Administration (FDA) is a CBD extract, in the form of Epidolex, for intractable pediatric epilepsies. However, anxiety, sleep problems, and stress are among the most common off-label uses of CBD (Moltke and Hindocha, 2021). CBD is non-intoxicating and abundantly produced in the hemp variety of *Cannabis sativa* L, which is typically classified as having less than 0.3% Δ^9^-THC (Nahler, 2019). The perception that CBD is safe (Kaur Bhamra et al., 2021) has even led to the off-label administration of CBD to children for treating numerous conditions including anxiety, hyperactivity, and autism spectrum disorder (ASD) (Poleg et al., 2019). These symptoms may derive from similar etiologies that could be targeted by a single treatment approach. Indeed, 30% of patients with ASD also have epilepsy which increases severity of additional comorbid symptoms that involve anxiety, sleep, and locomotor disturbances (Gillberg and Billstedt, 2000). Therefore, CBD may reduce symptoms associated with ASD (da Silva Junior et al., 2021), not to reduce neurodiversity, but to improve daily functioning and quality of life.

ASD is a complex neurodevelopmental disorder defined by core deficits with ranging severities in social, locomotor, and communicative behaviors (Fusar-Poli et al., 2020). Reduced GABAergic signaling (Coghlan et al., 2012; Cellot and Cherubini, 2014) and low levels of the endocannabinoid, anandamide (Karhson et al., 2018), have been implicated in the etiology of ASD symptoms. Boosting GABAergic signaling in preclinical mouse models of ASD (Yizhar et al., 2011; Han et al., 2012; Kaplan et al., 2017) or elevating anandamide signaling through inhibition of its degrading enzyme, FAAH (Kerr et al., 2016; Wei et al., 2016), rescues core social deficits. Initial clinical studies of CBD-rich cannabis treatment in human patients with ASD focused exclusively on comorbid symptoms but demonstrated promising effects (Aran et al., 2018; Barchel et al., 2019). Sublingual consumption of a 20: 1 CBD: Δ^9^-THC oil led to considerable behavioral improvement on the Clinical Global Impression of Change scale in 61% of patients (Aran et al., 2018). These improvements were accompanied by reduced anxiety levels, less frequent disruptive behavior, and improved communication. In a separate study, CBD-rich cannabis improved symptoms relating to hyperactivity, rage attacks, self-injurious behavior, sleep impairment, and anxiety (Barchel et al., 2019). Notably, 75% of patients reported improvement following treatment whereas symptoms only worsened in 4%. The most recent clinical study of CBD-rich cannabis was conducted in 60 5–11 year old children and found substantial benefits on social interaction (da Silva Junior et al., 2022) making it the first clinical investigation to assess CBD’s effect on a core ASD symptom. Additional improvements were observed in psychomotor agitation and food intake. The rates of symptom improvement reported in these studies are consistent with traditional prescription medications used in ASD therapy (McCracken et al., 2002; Marcus et al., 2009; Nadeau et al., 2011; Rossignol and Frye, 2014; Sturman et al., 2017), highlighting the potential of a cannabis-based treatment approach as a monotherapy.

Despite the promising outcomes emerging from the clinical trials, there are several limitations to which preclinical investigation will be able to inform clinical use of CBD-based treatment strategies in ASD. First, current clinical investigations have used cannabinoid formulations either not currently commercially available or prohibitively expensive (Elliott et al., 2020). Understanding the efficacy of commercially available products may help make cannabis-based treatment approaches more financially attainable for some families. Second, while CBD may be efficacious in the current trials’ formulations, its efficacy and effective dose range may be enhanced through the additive or synergistic actions of cannabis-derived volatile organic compounds such as terpenes (Russo, 2011, 2019).

The recognition that volatile organic compounds can have therapeutic properties stems back to around 3,000 BC with the first recorded description of aromatherapy (Hedaoo and Chandurkar, 2019). Essential oils used in aromatherapy may have calming, anxiolytic, and even pain-reducing properties under certain conditions (e.g., Navarra et al., 2015). These essential oils are usually comprised of multiple volatile compounds, similar to the terpene blends tested in this study, and it’s unclear whether their benefits derive from the action of a single compound in the oil or the coordinated action of many.

Preclinical models provide a platform for systematically examining how phytocannabinoids (e.g., CBD) and cannabis-inspired terpenes (e.g., formulated blends found in the OG Kush variety containing myrcene, limonene, and β-caryophyllene among other volatile organic compounds) impact behavior alone or in combination. Here, we tested the efficacy of vaporized CBD isolate in commercially available hemp oil as well as common cannabis-influenced terpene blends, either alone or in combination, on core social deficits and anxiety-related behavior in the well-defined BTBR mouse model of ASD (McFarlane et al., 2008). We hypothesized that passive inhalation of CBD rich vapor would induce prosocial effects and these effects would be enhanced by the addition of cannabis inspired terpenes. Our results reveal that both CBD and terpene blends inspired by popular cannabis strains have prosocial effects, and together, lead to improved symptom management in BTBR mice.

## Materials and methods

### Animals

BTBR *T*^+^ *Itpr3^tf^/*J (BTBR; Jackson Laboratories, Bar Harbor, ME) litters were bred in-house at Western Washington University. A total of 150 mice (18 males, 132 females) were used in these experiments. Mice were raised in standard laboratory housing in groups of 3–5 mice per cage on a 12 h light/dark cycle (lights on at 0700). Food and water were provided *ad libitum*. Mice were handled and habituated to the experimenter for a minimum of 5 min/day for 3 days prior to experimental assessment. All drug exposures and behavioral testing were conducted during the light cycle. All procedures conform to the regulations detailed in the National Institutes of Health *Guide for the care and use of laboratory animals* and were approved by the Institutional Animal Care and Use Committee at Western Washington University.

### Terpenes and CBD vape oils

Three commercially available unflavored CBD isolate-containing hemp oils were initially tested for CBD content (see details below): Savage Vape Shot (Savage Enterprises, Irvine, CA), Koi (Koi CBD, Norwalk, CA), and Blue Moon (Blue Moon Hemp, Pompano Beach, FL), each with a declared 1,000 mg of CBD isolate per 30 ml bottle. Terpene blends (i.e., OG Kush, Blue Dream, and Do-Si-Dos) and monoterpenes (i.e., β-caryophyllene, myrcene, and D-limonene) were gifted from Abstrax Tech (Tustin, CA). See Table 1 for composition details. Savage Vape Shot was used for all experiments when CBD oil was indicated (actual CBD concentration: 24.26 mg CBD/ml). CBD in Savage Vape Shot was CBD isolate with a 70/30 vegetable glycerin/propylene glycol base. When indicated, Savage Vape Shot or the terpenes were diluted in a vehicle solution comprised of 70% vegetable glycerin, 30% propylene glycol purchased from La Jolla Alcohol Research, Inc. (La Jolla, CA). The terpene blend concentration in vape oil was diluted to 5%. Terpene concentration was determined from the concentration found in each blend: D-limonene makes up 20% of the OG Kush blend, and therefore, was diluted to 1%; myrcene makes up 35% of the Blue Dream blend, and therefore, was diluted to 1.75%; β-caryophyllene makes up 24% of Do-Si-Dos, and therefore, was diluted to 1.2% with vehicle. Vape oil dilutions were prepared on the day of experiments.

**Table 1:** Concentration of volatile organic compounds in each blend.

Volatile organic compound	Concentration (%)
*OG Kush*	
D-Limonone	20
β-Caryophyllene	19
Myrcene	15
Linalool	12
Humulene	6
β-pinene	4
α-bisabolol	3
Fenchyl Alcohol	3
Terpineol	2
Caryophyllene oxide	2
*Blue Dream*	
Myrcene	35
α-pinene	24
β-Caryophyllene	14
β-pinene	12
D-Limonone	6
α-bisabolol	3
Humulene	2
Linalool	2
Terpineol	<1
Fenchyl Alcohol	<1
*Do-Si-Dos*	
β-Caryophyllene	24
D-Limonone	16
Myrcene	9
Humulene	9
Linalool	7
β-pinene	4
α-bisabolol	2
Fenchyl Alcohol	2
α-pinene	1
Terpineol	1

### Drug administration

Four 36 cm × 27 cm × 23 cm (L × W × H) ~ 17 L passive vapor inhalation chambers (La Jolla Alcohol Research, Inc) were programed to deliver precise vapor pulls for 6 s every 5 min for 30 min (starting at time point 0 for a total of 6 pulls per session; see Supplementary Figure 1). A consistent unidirectional airflow was created by a vacuum pump that pulled air and vapor through the chambers at a rate of 7.5 L/min. Each 6 s vapor pull draws 83.3 microliters of vape oil and leads to an approximately 2-min exposure (120.25 ± 4.55 s) to the vapor as it gets pulled through the chamber. The air intake port in the front of each chamber was connected to an air flow meter and tubing connected to a commercial SMOK TFV8 Baby Beast Tank with a 0.4 Ω atomizer coil (40–60 W range) filled with the prepared vape oil. Vapor pulls were computer controlled, which would send an electrical current to the base of the atomizer and delivered through the air intake port. Chamber air was then pulled through the chamber and passed through an in-line Whatman HEPA-Cap filter (Millipore-Sigma, St. Louis, MI). The air in the chambers appeared visibly clear of vapor prior to subsequent vapor pull. Since the vapor gets evenly distributed across 4 chambers, each 6 s vapor pull leads to the delivery of 0.51 mg of CBD in the undiluted CBD product.

### Behavioral assessment

Behavioral assessments began between postnatal day 80 and 200. All experiments were run as a repeated measures design, expect for the elevated plus maze for which ages were counterbalanced across conditions. Exposure conditions were counterbalanced for all experiments. Each experiment included subjects from a minimum of two litters. Animals are removed from the chambers 5 min after the last vapor exposure and are then moved to the behavioral room. Animal behavior was tested approximately 20 min following the last vapor exposure. Animal movement was recorded in the presence of overhead fluorescent light using a digital camera (Microsoft LifeCam) mounted above the behavioral apparatus. Behavior was analyzed using ezTrack open source animal tracking software (Pennington et al., 2019). Each video was checked for accurate assessment by visually inspecting output bokeh plots and calculating total ratios to ensure that 100% of their behavior was captured in analysis. At the end of each trial, the behavioral apparatus was cleaned with 70% ethanol and wiped with paper towels.

### Three chamber test of social interaction

Experiments were conducted as a within-subjects design and exposure conditions were counterbalanced between subjects. The apparatus is a nontransparent Plexiglas box (58 cm × 30 cm) with two partitions that make left, center, and right chambers (30 cm × 19.3 cm). Each partition has a square opening (5 cm × 5 cm) in the bottom center. Inverted cylindrical wire cages (10.5-cm diameter; Galaxy Pencil Cup; Spectrum Diversified Designs) were placed in opposite corners of the chamber (top left and top right) and were used as an inanimate object or to cage the stranger mouse. Cylindrical bottles filled with water were placed on top of the wire cups to prevent the test mouse from climbing on top of the cups. The wire cups and chamber were cleaned with 70% ethanol and wiped with paper towels between each test mouse. In the habituation phase, a test mouse was placed in the center of the chamber without wire cups and allowed to freely explore the three chambers for 10 min. For each experiment, mice did not show a side preference during the habituation period (all *p* > 0.05). Locomotor activity was also measured during the habituation phase and compared across conditions in a between-subjects manner for only the first round of each experiment to eliminate any practice effects on exploratory locomotor activity. After habituation, the test mouse was then returned briefly to its home cage. For the test phase, a stranger age-and sex-matched C57BL/6 J mouse (Jackson Laboratories, Bar Harbor, ME) was placed in one of the two wire cups; the opposite wire cup was empty. The test mouse was then returned to the center of the chamber and allowed to freely explore for 10 min. The side of the chamber with the stranger mouse was counterbalanced between trials. Time spent within a 5-cm radius proximal to each wire cage was measured and recorded as time interacting with the “social” or “object” stimulus. A social preference was defined as a statistically-significant preference for engaging in social interaction as a function of the total interaction (time spent interacting with the social stimulus and non-social, “object,” stimulus): 0.5 > (interaction with social stimulus/total stimulus interaction).

### Elevated plus maze

These experiments were conducted as a between-subjects design to prevent practice effects. Ages were counterbalanced across conditions. Subjects were placed in the center of the white plus-shaped maze and allowed to explore for 5 min. Each of the 4 maze arms is 60 cm × 6 cm connected in the middle at a 6 cm × 6 cm open center (total 126 cm in length). Two “closed” arms are surrounded by 21 cm opaque plexiglass walls on 3 sides while the other two “open arms” are open on all sides. The maze is elevated 93 cm above the floor. The ratio of time spent in the open arms/closed arms was assessed using ezTrack. Head dip frequency, grooming frequency, and grooming duration were assessed over video by an observer blind to the experimental condition. Experimenters left the behavioral room during the experiment and monitored behavior on a computer monitor through a narrow window. The EPM test was conducted with full overhead lighting.

### Quantification of CBD in commercial products by LC/Q-TOF

We used an LC-QTOF-MS system to quantify CBD in our samples, specifically an Agilent 1290 UHPLC with an AdvanceBio 6545 XT Q-TOF. Separation was attained with an Agilent Eclipse+ C18 RRHD column, a 0.2 ml/min flow rate, and a 10-min gradient transitioning between water with 0.1% formic acid (solvent A) and acetonitrile (solvent B), see Table 2. The system was fitted with an electrospray source with the capillary voltage and nozzle voltage set at 3500 and 2000 V, respectively. Within the mass spectrometer the fragmentor voltage was set at 175 V while the skimmer was at 60 V. Analyte confirmation and peak integration was completed with Agilent MassHunter software.

**Table 2:** Solvent gradient used by LC-QTOF-MS to determine CBD quantity.

Time (min)	A (%)	B (%)
0	70	30
1	70	30
6	5	95
9	5	95
9.1	70	30
10	70	30

Solvent A was 0.1% formic acid. Solvent B was acetonitrile.

### Quantification of CBD in plasma by LC/Q-TOF

Male and female BTBR mice (*n* = 4 male and *n* = 4 female) were exposed to undiluted Savage Vape Shot for the same 30-min protocol as used throughout the experiments (i.e., 6 s vapor pulls every 5 min for 30 min). Immediately after the 30-min exposure period, blood was collected by cardiac puncture and placed in lithium heparin BD Mircotainer tubes (Becton Dickson, NJ, USA). The blood sample was then centrifuged at 2,000 rcf (g) for 10 min at 4°C. Serum plasma was then transferred to a separate tubes for the liquid–liquid extraction procedure. Here 100 uL of plasma was added to 200 uL of acetonitrile and vortexed for 60 s. 50 mg salt mixture containing a 4:1:1 ratio of magnesium sulfate, sodium chloride, and sodium citrate was added and vortexed for another 60 s. The samples were then centrifuged at 10,000 rcf (g) for 10 min at 4°C. The plasma was then extracted and stored at −80°C until testing.

For the quantification of CBD in mouse plasma, the same instrument was used as was detailed previously for the CBD oil concentration verification in commercial products. The system, column, solvents and flow rate remained the same. To achieve separation of this more complex sample matrix an extended gradient was used, see Table 3. The system was fitted with an electrospray source with the capillary voltage and nozzle voltage set at 3000 and 1,500 V, respectively. Within the mass spectrometer the fragmentor voltage was set at 100 V while the skimmer was at 60 V. Analyte confirmation and peak integration was completed with Agilent MassHunter software. It was known that the sample concentrations would be low and near the detection limit, so a larger injection volume was used to concentrate the sample within the instrument. This concentration factor as well as the dilution done during the liquid–liquid extraction was used to calculate the final dilution factor of 0.2, which was used calculate the final sample concentration.

**Table 3:** Solvent gradient used by LC-QTOF-MS to determine CBD quantity in plasma.

Time (min)	A (%)	B (%)
0	95	5
2	50	50
8	20	80
9	5	95
10	5	95
10.5	95	5
12	95	5

Solvent A was 0.1% formic acid. Solvent B was acetonitrile.

### Statistical analysis

All data are shown as mean ± S.E.M. and analyzed by either one-way ANOVA, repeated measures ANOVA, or paired t-tests where appropriate using Sigma Plot software (SPSS Inc) with an alpha set at 0.05, all tests two-tailed. For analyzing the elevated plus maze data, we used a Kruskil-Wallis ANOVA on ranks due to a statistically significant Shapiro–Wilk test suggesting we violated the assumption of a normal distribution of data (*p* < 0.05). Tukey’s HSD *post hoc* comparisons were used to analyze main effects and interactions. Descriptive statistics (mean ± S.E.M.), as well as the number of subjects and litters used in each experiment are included in Supplemental Table S1. For all figures, * indicates *p* < 0.05; ** indicates *p* < 0.01; *** indicates *p* < 0.001.

## Results

Because of the increasing popularity of commercially available CBD-rich hemp products (Leas et al., 2019; Williamson et al., 2020), we assessed the efficacy of a commercially available CBD-rich hemp extract (see methods for product details). Since the declared CBD content on the product label of commercial CBD hemp products is often inaccurate (Johnson et al., 2022; Miller et al., 2022), we first sought to identify the CBD concentration in three hemp samples that all claimed a concentration of 33.3 mg/ml. Analysis of CBD concentration using LC/Q-TOF revealed that CBD concentrations were lower than reported in all three samples and ranged from 21.32 mg/ml to 24.26 mg/ml (Supplementary Figure 2). For our behavioral assessment, we chose to use the Savage Vape Shot product because it had the highest CBD concentration.

**Figure 1:**
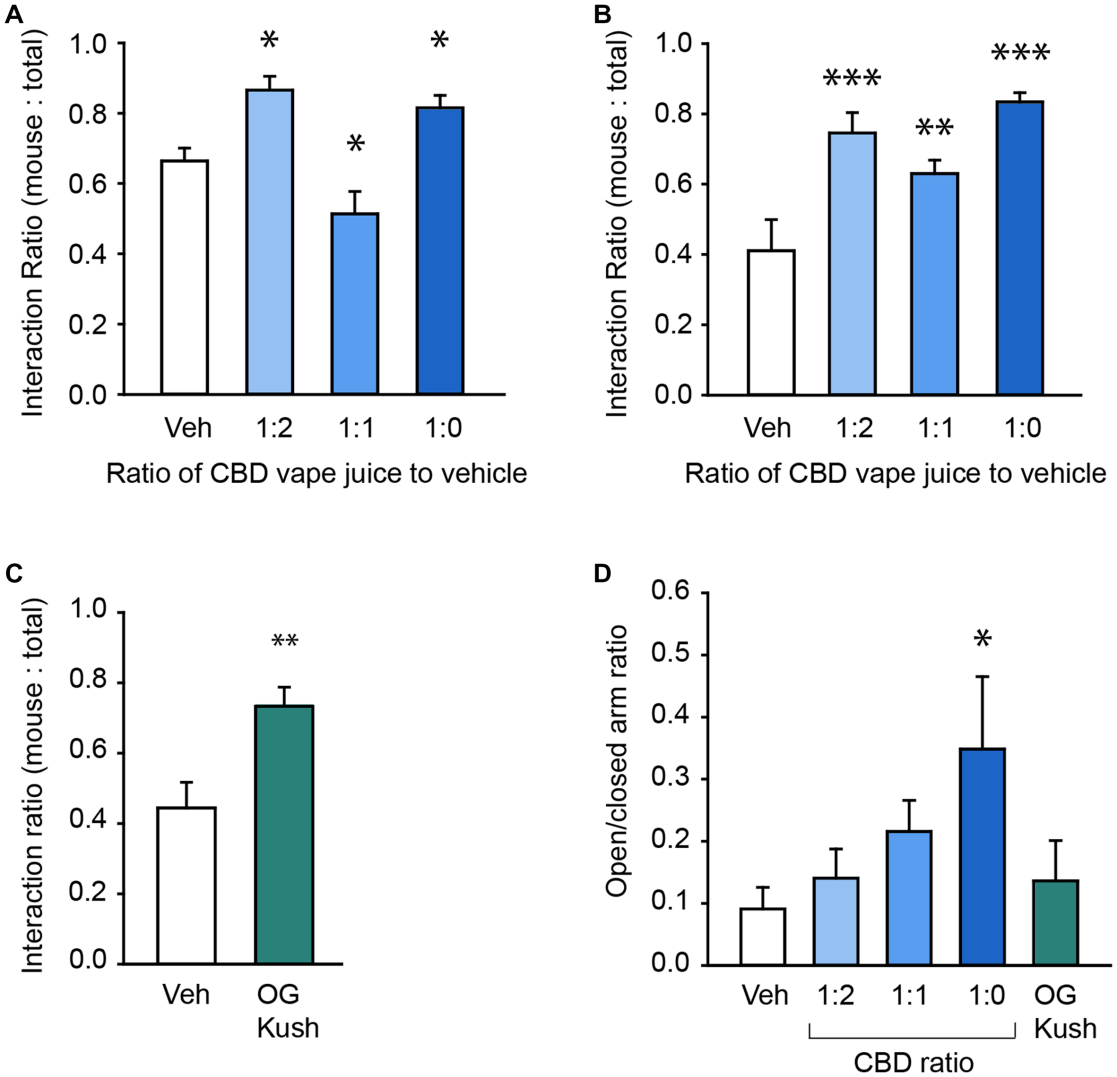
CBD and OG Kush terpenes have prosocial effects. **(A)** Bar chart showing the effect of vehicle and four different CBD oil concentrations on the social interaction ratio in the Three Chamber Test of Social Interaction in male mice. **(B)** Bar chart showing the effect of vehicle and four different CBD oil concentrations on the social interaction ratio in female mice. **(C)** Bar chart showing the effect of the OG Kush terpene blend on the social interaction ratio in female mice. **(D)** The effect of different CBD oil ratios and OG Kush terpenes on the ratio of time spent in the open versus closed arms of the elevated plus maze in female mice. * Indicate differences compared to the vehicle condition. **p < 0.01; ***p < 0.001.

To test CBD’s efficacy in treating core ASD-like social deficits in our BTBR mice, we used the 3-Chamber Test of Social Interaction (Crawley, 2012) and measured the effect of vaporized CBD oil at four different concentrations (vehicle, 1 part CBD oil to 2 parts vehicle [1:2], 1 part CBD oil to 1 part vehicle [1:1], and undiluted CBD oil [1,0]) on the ratio of time spent interacting with a novel stranger mouse compared to the total time in interaction with either the mouse or an inanimate object in male and female mice. Each 6 s vapor pull of the undiluted CBD oil delivered 0.51 mg of CBD into each chamber (see Method section for details). By the end of the 30-min exposure session, in which mice were exposed to six, six second vapor pulls, plasma CBD concentrations were 0.13 ± 0.02 ng/ml (range: 0.08–0.19 ng/ml; Supplementary Figure 3). A two-way repeated measures ANOVA revealed a main effect of exposure condition, *F*(3,42) = 17.57, *p* < 0.001, and an interaction between mouse sex and CBD concentration, *F*(3,42) = 5.03, *p* = 0.005, on the social interaction ratio (Figure 1; Table 3). In male mice, both the diluted 1:2 ratio and undiluted 1:0 ratio hemp oil increased the social interaction ratio, whereas the 1:1 ratio reduced the social interaction ratio compared to vehicle treatment (all *p* < 0.05, Figure 1A). In female mice, all 3 CBD concentrations increased social interaction, all *p* < 0.01, Figure 1B) without affecting locomotor activity, *p* = 78 (Supplementary Figure 4). Notably, males and females differed in their social interaction ratios in the vehicle condition, *p* = 0.001. In contrast to what we predicted based on previous reports (Yang et al., 2012), one-sample *t*-tests revealed that male BTBR mice showed a significant social interaction preference, *t*(7) = 4.54, *p* = 0.003, whereas female mice did not, *p* = 0.4. We replicated this prominent social preference in a separate cohort of male mice, *t*(5) = 4.36, *p* = 0.004. Therefore, we focused the rest of our behavioral assessment to female mice to further investigate pharmacological strategies that impact social deficits and related comorbidities.

While CBD oil caused prosocial effects in our BTBR strain, it’s unclear if cannabis terpenes also contribute therapeutic benefits on their own and may be responsible for the high degree of efficacy reported anecdotally from whole-plant preparations. We started by testing the effect of vapor delivery of a blend of terpenes from the OG Kush cannabis strain (5% OG Kush terpenes, 95% vegetable glycerin/propylene glycol vehicle) on social behavior in the 3-Chamber Test. Like CBD, a paired t-test revealed that OG Kush terpenes caused a robust increase in the social interaction ratio compared to vehicle, *t*(8) = 3.69, *p* = 0.006 (Figure 1C). Interestingly, a Kruskal-Wallis one-way ANOVA on ranks identified that only the undiluted CBD oil, but not other concentrations, nor OG Kush terpenes, reduced general anxiety on the elevated plus maze, *H*(4) = 12.25, *p* = 0.02 (Figure 1D). There were no impacts of any of the CBD concentrations nor OG Kush on head dips, grooming frequency, or time spent grooming (all *p* > 0.05; Supplementary Figure 5). This suggests that some of CBD and OG Kush’s prosocial benefits are independent of reducing general anxiety since changes in social interaction behavior was observed without changes in general anxiety.

**Figure 2:**
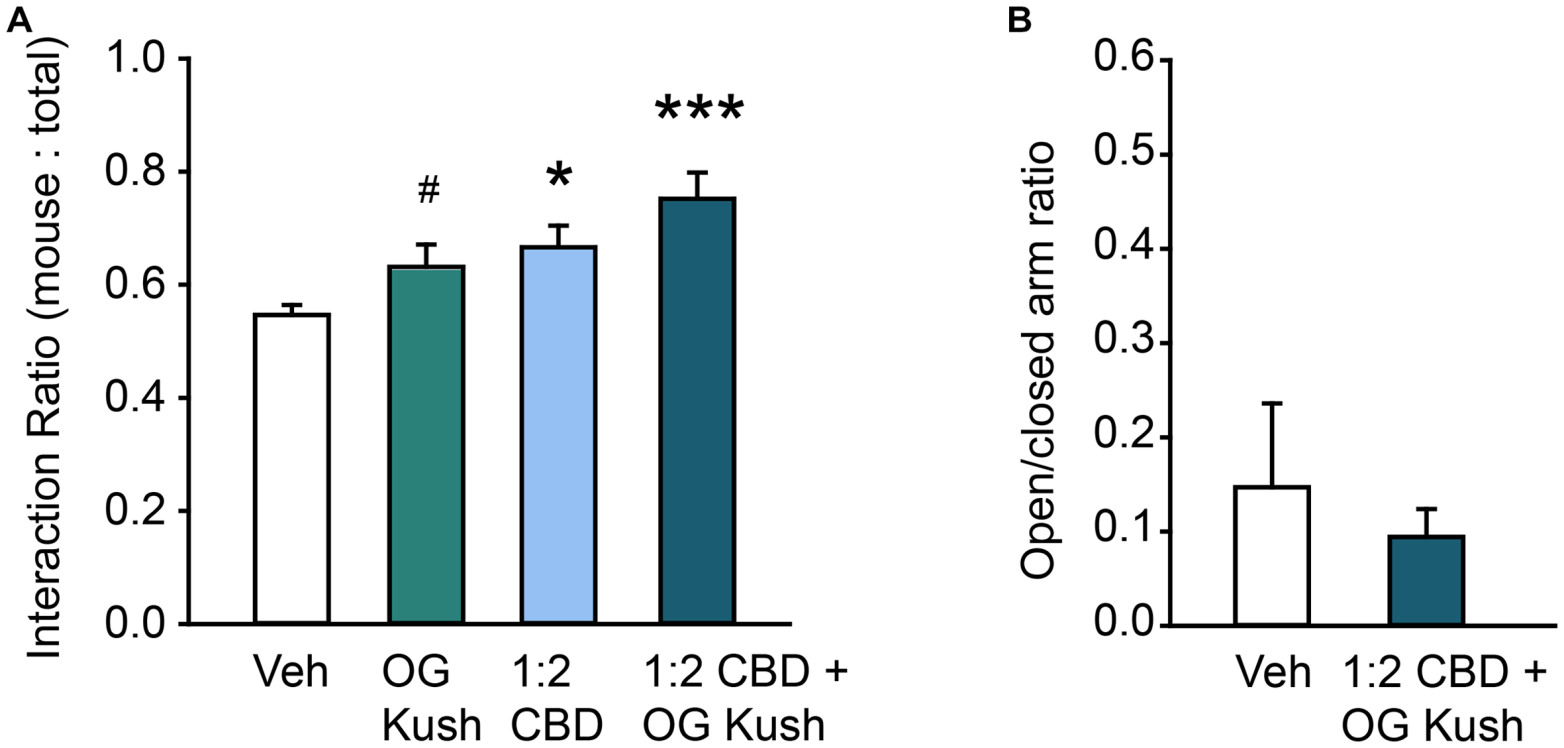
Combination of CBD oil and OG Kush terpenes have robust prosocial effects. **(A)** Bar chart showing the effect of OG Kush terpenes, CBD oil, or the combination on the social interaction ratio in the Three Chamber Test of Social Interaction in female mice. The combination and presumably the relative ratio of these volatile organic compounds is important for their prosocial effects. **(B)** Bar chart showing the effect of a ratio of 1:2 CBD to vehicle plus OG Kush terpenes on the ratio of time spent in the open versus closed arms of the elevated plus maze in female mice. * Indicates difference compared to the vehicle condition following Tukey’s posthoc comparisons; # indicates difference compared to vehicle from *a priori* paired contrasts, *p* < 0.05. ***Indicates difference compared to vehicle condition following Tukey’s posthoc comparisons, *p* < 0.001.

We next tested the hypothesis that a combination of CBD oil with added terpenes would be more efficacious than the two components on their own. This is often referred to as the “Entourage Effect,” and although it has strong theoretical basis (Russo, 2011, 2019), the impact that innovative combinations of terpenes and cannabinoids have on various conditions, including social behavior in ASD, are largely understudied (Ferber et al., 2020). We therefore retested the effect of a 1:2 CBD oil: vehicle solution and 5% OG Kush terpenes, alone and in combination on prosocial behavior in the 3-Chamber Test. *A priori* pairwise comparisons supported the replication of our earlier results that both a 1:2 CBD oil:vehicle solution and 5% OG Kush terpenes, independently increased the ratio of time spent in social interaction compared to vehicle (all *p* < 0.05). A one-way repeated measures ANOVA with Tukey’s posthoc comparisons found a main effect of exposure condition on social behavior, *F*(3,36) = 7.66, *p* < 0.001, and the combination of 1:2 CBD oil combined with 5% OG Kush terpenes had the most robust prosocial effect (*p* < 0.001; Figure 2A). These findings suggest that a combination of CBD oil and OG Kush terpenes leads to stronger and more robust prosocial benefits. These prosocial effects were independent of changes to general anxiety as the combination of OG Kush and 1:2 CBD oil had no impact elevated plus maze performance, *p* = 0.58 (Figure 2B).

**Figure 3:**
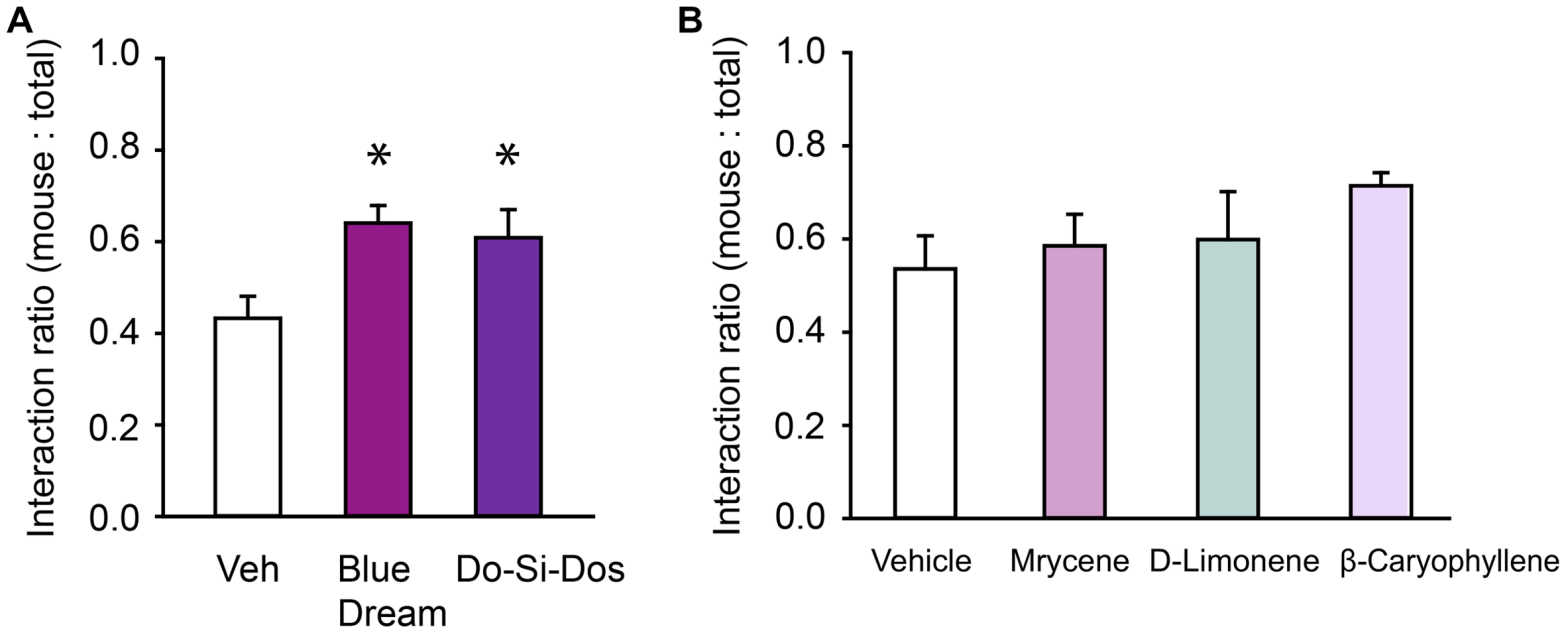
Cannabis terpene blends confer prosocial effects. **(A)** Bar chart showing the effect of Blue Moon and Do-Si-Dos terpene blends have on the social interaction ratio in the Three Chamber Test of Social Interaction in female mice. **(B)** Bar chart showing the effect of individual terpenes on the social interaction ratio in female mice. * Indicates differences compared to the vehicle condition.

The prosocial effects we observed with fresh OG Kush terpenes prompted investigation of the potential prosocial effects of other cannabis terpene blends from common strains such as Do-Si-Dos and Blue Dream. Each blend’s composition of volatile organic compounds is listed in Table 2. Similar to OG Kush, a repeated measures ANOVA found that both Blue Dream and Do-Si-Dos terpene blends (5% terpenes, 95% vegetable glycerin/propylene glycol vehicle) increased the social interaction ratio, *F*(2,14) = 4.56, *p* = 0.03 (Figure 3A). Together, these findings demonstrate that cannabis terpene blends can contribute to the prosocial benefits in ASD and highlight the benefits of a commercially available hemp oil containing CBD isolate. We hypothesized that it was the unique blends of volatile organic compounds, and not a single terpene within the blend, that conveyed the prosocial effects we observed. To test this hypothesis, we assessed each terpene blend’s most abundant terpene, alone and at the concentration found in each blend on social interaction behavior. Since each terpene blend was tested at a concentration of 5%, the following terpene concentrations were tested to match the individual terpene concentration from each blend: 1% D-Limonene (most abundant in OG Kush), 1.5% β-caryophyllene (most abundant in Do-Si-Dos), and 1.75% myrcene (most abundant in Blue Dream). Consistent with our hypothesis, a one-way repeated measures ANOVA did not reveal any effect of the individual terpenes on the social interaction ratio, *F*(3,18) = 1.20, *p* = 0.34 (Figure 3B). Therefore, our results suggest that the most abundant terpene in each blend is not solely responsible for the prosocial benefits we observed from the complete blends. Instead, the unique combination of volatile organic compounds in each blend are important in promoting prosocial behavior.

## Discussion

Numerous anecdotal cases and accumulated caregiver reports suggest that CBD can reduce core symptoms of ASD and improve quality of life (Barchel et al., 2019; Bar-Lev Schleider et al., 2019). Several early clinical trials have found that CBD can improve comorbid symptoms of ASD (Aran et al., 2018; Barchel et al., 2019), but only one assessed and found benefits on core social interaction behaviors (da Silva Junior et al., 2022). These studies used CBD-rich cannabis oils that contain a 1:20 ratio of Δ^9^-THC to CBD, more than the 0.3% limitation of Δ^9^-THC to legally classify as “hemp,” and therefore, may make it increasingly difficult for patients to access because of legal restrictions or prohibitive cost. Safety concerns among caregivers with administering Δ^9^-THC to children may also limit this formulation’s utility. This and other medicinal cannabis formulations tend to focus exclusively on phytocannabinoids (e.g., CBD and Δ^9^-THC) and fail to consider the potential therapeutic benefits conferred by terpenes. An empirical understanding of the effects that common terpene blends have on ASD symptoms could lead to the development of safer, more effective, and more accessible treatment options. Our findings suggest that combining cannabis-inspired terpene blends with CBD may be an efficacious strategy for improving social behavior that avoids legal restrictions on THC levels and mitigates concern over administering THC to children and adolescents.

We tested the effect of a commercially available CBD-rich hemp oil along with several terpene blends from common cannabis strains on social interaction behavior in BTBR mice. Our results add further support to preclinical findings of CBD’s prosocial effects in ASD models (Kaplan et al., 2017; Mastinu et al., 2022) and the latest clinical trial (da Silva Junior et al., 2022). We also provide the first known evidence for the prosocial effects of terpene blends from popular cannabis strains. Our findings support four general conclusions: (1) a commercial hemp oil can have prosocial effects, (2) cannabis terpene blends confer their own prosocial effects and can lead to more robust prosocial effects when combined with CBD, and (3) the prosocial effects of cannabis terpene blends derive from the combination of multiple independent terpenes, and (4) the prosocial effects can be achieved independent of reductions in general anxiety. These findings should inform the development of novel phytocannabinoid and terpene compositions for treating symptoms of ASD, not to reduce neurodiversity, but to improve the quality of life for patients.

We observed prosocial effects from the inhalation of vaporized blends of terpenes found in popular cannabis strains, OG Kush, Do-Si-Dos, and Blue Dream. We tested if the single most abundant terpene in each blend was sufficient to have prosocial effects, or whether the combination of terpenes was needed. Since the most abundant terpenes, at the concentration and delivery dose administered for the blends, did not meet the threshold for increasing social interaction, we conclude that the combination of multiple volatile compounds found in the OG Kush, Do-Si-Dos, and Blue Dream terpene blends is important for reliably conferring the prosocial effects we observed. However, since we only tested a single terpene from each blend, it is possible that other constituents in the blends that were expressed at lower concentrations may have conferred its prosocial effects. Other benefits have been observed with individual terpenes such as reduced anxiety (Malcolm and Tallian, 2017), dampened pain (Klauke et al., 2014), and improved mood (Ferber et al., 2020), but their effects on prosocial behavior in ASD had not been assessed until this study. Our findings suggest that, at least in the BTBR mouse model of ASD, unique combinations of these terpenes lead to more robust prosocial effects than individual terpenes alone.

Another main finding is that the combination of diluted CBD oil and OG Kush terpenes had robust prosocial effects in the Three Chamber Test. We planned this experiment to be a test of the Entourage Effect Hypothesis which posits that the combination of multiple phytochemicals can improve the efficacy of a single one (Ben-Shabat et al., 1998). Our observation that the combination of CBD and OG Kush terpenes led to more robust prosocial effect than either component on their own supports this hypothesis. Based on the prosocial effects of both CBD and OG Kush terpenes, independently, the robust prosocial benefit of the combination is consistent with additive effects, as opposed to synergistic potentiation of two sub-therapeutic doses. The prosocial benefits stemming from the combination of CBD and terpenes supports the hypothetical, but previously untested, assertion that prososocial efficacy can be enhanced by the combination of terpenes and phytocannabinoids, which may be a safer effective alternative than adding THC. Like combinatorial benefits of adding OG Kush to CBD, we found that two additional cannabis-inspired terpene blends, Do-Si-Dos and Blue Dream, had prosocial effects, but the three most abundant terpenes in each did not improve sociability more than vehicle. Therefore, the combination and presumably the relative ratio of these volatile organic compounds is important for their prosocial effects. Together, these findings highlight that combinations of phytochemicals can lead to enhanced therapeutic benefits than individual chemicals.

At this point, our conclusions only pertain to female mice. Although we observed that CBD had prosocial effects in both males and females, we failed to observe a baseline social interaction deficit in males in two separate cohorts of male BTBR mice. This was surprising given that BTBR mice are a commonly used mouse model of ASD (McFarlane et al., 2008) and male mice are more commonly tested than females. While male subjects have historically dominated pre-clinical research, it’s especially prevalent in ASD research (e.g., Pearson et al., 2011), which is often justified by the higher prevalence of males than females with ASD. However, given the 1: 3 ratio of females to males with ASD (Loomes et al., 2017), females certainly warrant investigation as well. Most reports describe a robust social interaction deficit among male BTBR mice in the Three Chamber Test of Social Interaction, whereas female BTBR mice are less consistent in their social deficit phenotype because of their enhanced sensitivity to different characteristics of the stimulus mice (Meyza et al., 2012). In our hands, female BTBR mice displayed a consistent lack of social preference in the Three Chamber Test whereas males initially did not. The exact reasons for this are unclear. BTBR mice have an exaggerated response to stress (Benno et al., 2009), especially for novel social situations (Pobbe et al., 2011), and so it’s possible that the male mice experienced some unique interaction of these factors during their testing period that promoted a social preference. Another possibility is that the repeated periods of discrete inhalation periods of the vehicle vapor affected their olfactory processing that disrupted their social sensory cues in a way that facilitated more interaction time, perhaps by requiring longer sniff durations which are normally shorter in male BTBRs (Yang et al., 2012). Future experiments should seek to understand the impacts of terpene blends in males to identify if the therapeutic utility in females can extend to both sexes since sex differences in the response to volatile organic compounds have been reported across a number of phenotypes including anxiety (Bradley et al., 2007), pain (Ceccarelli et al., 2004), and neurotransmitter release (Ceccarelli et al., 2002).

One of this study’s limitations is that estrus cycle was not controlled for in female subjects. Recent evidence highlights the importance of estrus cycle for interpreting female mouse social behavior (Chari et al., 2020). Social behavior may be particularly elevated during estrus due to enhanced excitability of midbrain dopamine neurons from increased estradiol levels (Shanley et al., 2023). However, these assessments are commonly conducted in C57BL/6 mice who typically display high social preference. The impact of estrus cycle on female social behavior in BTBR mice is not well-documented. Given the importance of estrus cycle on social behavior in other mouse strains, future investigations assessing the interaction between estrus cycle phase and the prosocial efficacy of CBD and terpenes are warranted.

One of this study’s strengths is that we administered CBD and terpenes *via* discrete pulls of vaporized oils. Vapor inhalation better models human consumption patterns of cannabis (Aston et al., 2019; Lim et al., 2022) and more closely matches the pharmacokinetic parameters of sublingual/oromucousal absorption (Millar et al., 2018) used in human studies of CBD’s effect on ASD symptoms (Aran et al., 2018; Barchel et al., 2019). However, given the notable variability in blood drug levels following passive drug inhalation (MacLean et al., 2017), we sacrificed precise dose control obtained with injection methods to better model the use and pharmacokinetic parameters relevant for ASD treatment. This lack of precision is illustrated in the fair amount of variability in plasma CBD concentrations we measured. Notably, our observed plasma CBD concentration range is quite low compared to those achieved for treating other disorders. This highlights that small amounts of CBD may be effective for some behavioral symptoms but not others. For instance, on the lower end, plasma CBD concentrations of 4.7–17 ng/ml were associated with reduced neural responses to threatening faces in humans (Fusar-Poli et al., 2009). On the higher end, antiepileptic CBD plasma concentration often build to several hundred ng/ml following several weeks of daily dosing to achieve maximal clinical efficacy (Szaflarski et al., 2019). These differences in the therapeutic plasma levels for epilepsy and other disorders can be quite drastic, especially in the case of comparing CBD’s effects in ASD compared to epilepsy where effective doses for treating ASD can be 71 times lower (Bilge and Ekici, 2021) than for epilepsy (Szaflarski et al., 2019). This has been confirmed in the *Scn1a^+/−^* mouse model that shares both epilepsy and ASD-like social impairment phenotypes where the prosocial benefits were found at 1/10th that of the antiepileptic dose (Kaplan et al., 2017). Unfortunately, the minimal prosocial dose threshold was never determined that may otherwise have corresponded to the prosocial benefits associated with low plasma levels observed in this study, which we reveal here to be relatively low. The prosocial benefits of low-dose CBD disappear with higher doses (Kaplan et al., 2017), consistent with the common inverted-U dose–response curve of CBD’s therapeutic efficacy (Guimarães et al., 1990; Zuardi et al., 2017). This highlights the dosing challenge when trying to treat multiple symptoms simultaneously, such as social behavioral in ASD and seizures in epilepsy which may be comorbid in approximately 30% of cases (Tye et al., 2018). Integrating additional chemicals to CBD, such as the terpene blends studied here, may extend this therapeutic dose range to achieve symptom control across several conditions.

There is debate over the necessity of olfactory stimulation to experience the therapeutic benefits of volatile compounds, such as cannabis terpenes, as several have been shown to act directly on neurotransmitter systems. For instance, β-caryophyllene activation of cannabinoid type II receptors (Gertsch et al., 2008) contributes to its anti-inflammatory and pain-relieving properties (Klauke et al., 2014). Additionally, linalool and some of its metabolic products enhance GABAergic currents, *in vitro* (Milanos et al., 2017). However, linalool’s direct targeting of limbic GABAergic signaling may not be its therapeutic mechanism since ablating the olfactory epithelium blocked its anxiolytic action in mice, thereby suggesting that olfactory stimulation is necessary to achieve its anxiolytic effects, at least (Harada et al., 2018). Whether olfactory stimulation also mediates the prosocial effects of the terpene blends or if they work directly on central signaling mechanisms downstream of the olfactory epithelium remains to be tested. Yet, this may be important for therapeutic efficacy of the terpene blends since the reliance on repeated bouts of olfactory stimulation, as achieved by our discrete vapor puff protocol, may not transfer to non-vaporization consumption methods (e.g., oral capsules) or experimental protocols (e.g., injection methods).

The reliance on olfactory stimulation for the terpene’s prosocial effects may also be impacted by one’s olfactory sensitivity. There is great olfactory heterogeneity among those with ASD for odor detection thresholds, identification (Larsson et al., 2017), and neural responses to odorant presentation (Xu et al., 2020). Those with ASD may show extreme effect sizes for hyposensitivity or hypersensitivity (Larsson et al., 2017). BTBR mice effectively discriminate both social and non-social odors but display lower-than average sniff times (Moy et al., 2007; Yang et al., 2012), thereby suggesting intact but somewhat abnormal olfactory processing. If olfactory stimulation by the terpenes is necessary for their prosocial effect, then terpene blend concentrations may need to be modulated depending on the individual’s olfactory sensitivity phenotype.

In conclusion, we present the first known evidence for the prosocial effects of cannabis terpene blends in a preclinical ASD model. Further, combining terpenes with CBD promotes more robust therapeutic benefits. These findings highlight the value of including cannabis terpenes in formulations being tested in human ASD clinical trials. Future studies should seek to validate these findings in males showing social deficits and continue to optimize CBD and terpene blends for improved efficacy.

## Data availability statement

The datasets supporting the conclusions in this study can be made available upon request to the corresponding author without undue reservation.

## Ethics statement

The animal study was reviewed and approved by Institutional Animal Care and Use Committee at Western Washington University.

## Author contributions

JS, MK, KR, and JK designed the experiments. JS, MK, KR, LG, and JM conducted behavioral testing and were involved in data analysis and interpretation. FM and SK were involved in cannabidiol product acquisition, testing, and interpretation. MK and SK were involved in plasma CBD concentration collection, testing, and interpretation. IO, KK, and TM created the cannabis-inspired terpene blends and helped interpret data. JK wrote the manuscript. All authors were involved in editing and revising the manuscript. All authors contributed to the article and approved the submitted version.

## Funding

Manuscript preparation grant through the Office of Research and Sponsored Programs from Western Washington University will support the open access publication fee.

## Conflict of interest

IO, KK, and TM are employees of Abstrax Tech, Inc.

The remaining authors declare that the research was conducted in the absence of any commercial or financial relationships that could be construed as a potential conflict of interest.

## Publisher’s note

All claims expressed in this article are solely those of the authors and do not necessarily represent those of their affiliated organizations, or those of the publisher, the editors and the reviewers. Any product that may be evaluated in this article, or claim that may be made by its manufacturer, is not guaranteed or endorsed by the publisher.
