# Supplementary material for: Cannabidiol and cannabis-inspired terpene blends have acute prosocial effects in the BTBR mouse model of autism spectrum disorder
Source: Front Neurosci. 2023 Jun 16;17:1185737. doi: 10.3389/fnins.2023.1185737 (PMC10311644; doi:10.3389/fnins.2023.1185737)
Supplement: Supplementary file 1 [file Table_1.docx]

Supplemental Table 1

| **Figure** | **Sex** | **Condition** | **Number of subjects** | **Descriptive statistics** |
| --- | --- | --- | --- | --- |
| 1a | M | Vehicle | 8 | 0.66 ± 0.04 |
| 1a | M | 1 : 2 ratio | 8 | 0.87 ± 0. 04 |
| 1a | M | 1 : 1 ratio | 8 | 0.51 ± 0.06 |
| 1a | M | 1 : 0 ratio | 8 | 0.82 ± 0. 04 |
| 1b | F | Vehicle | 8 | 0.41 ± 0.09 |
| 1b | F | 1 : 2 ratio | 8 | 0.75 ± 0.06 |
| 1b | F | 1 : 1 ratio | 8 | 0.63 ± 0.04 |
| 1b | F | 1 : 0 ratio | 8 | 0.83 ± 0.03 |
| 1c | F | Vehicle | 9 | 0.45 ± 0.07 |
| 1c | F | OG Kush | 9 | 0.73 ± 0.05 |
| 1d | F | Vehicle | 12 | mean: 0.09 ± 0.04; median: 0.07 |
| 1d | F | 1 : 2 ratio | 12 | mean: 0.14 ± 0.05; median: 0.10 |
| 1d | F | 1 : 1 ratio | 12 | mean: 0.22 ± 0.05; median: 0.14 |
| 1d | F | 1 : 0 ratio | 12 | mean: 0.35 ± 0.12; median: 0.15 |
| 1d | F | OG Kush | 12 | mean: 0.14 ± 0.06; median: 0.06 |
| 2a | F | Vehicle | 13 | 0.55 ± 0.02 |
| 2a | F | OG Kush | 13 | 0.63 ± 0.04 |
| 2a | F | 1:2 CBD | 13 | 0.67 ± 0.04 |
| 2a | F | OG Kush + 1:2 CBD | 13 | 0.75 ± 0.05 |
| 2b | F | Vehicle | 7 | 0.15 ± 0.09 |
| 2b | F | OG Kush + 1:2 CBD | 7 | 0.09 ± 0.03 |
| 3a | F | Vehicle | 8 | 0.43 ± 0.05 |
| 3a | F | Blue Moon | 8 | 0.64 ± 0.04 |
| 3a | F | Do-Si-Do | 8 | 0.61 ± 0.06 |
| 3b | F | Vehicle | 7 | 0.54 ± 0.07 |
| 3b | F | β-caryophyllene | 7 | 0.72 ± 0.03 |
| 3b | F | Myrcene | 7 | 0.59 ± 0.07 |
| 3b | F | D-limonene | 7 | 0.60 ± 0.10 |
| Supplemental Figure 5a | F | Vehicle | 12 | 8.42 ± 0.94 |
| Supplemental Figure 5a | F | 1:2 CBD | 12 | 7.25 ± 0.88 |
| Supplemental Figure 5a | F | 1:1 CBD | 12 | 8.40 ± 0.82 |
| Supplemental Figure 5a | F | 1:0 CBD | 12 | 7.67 ± 0.87 |
| Supplemental Figure 5a | F | OG Kush | 12 | 6.67 ± 0.83 |
| Supplemental Figure 5b | F | Vehicle | 12 | 3.08 ± 0.53 |
| Supplemental Figure 5b | F | 1:2 CBD | 12 | 3.00 ± 0.35 |
| Supplemental Figure 5b | F | 1:1 CBD | 12 | 2.30 ± 0.37 |
| Supplemental Figure 5b | F | 1:0 CBD | 12 | 2.58 ± 0.29 |
| Supplemental Figure 5b | F | OG Kush | 12 | 2.67 ± 0.40 |
| Supplemental Figure 5c | F | Vehicle | 12 | 90.17 ± 19.91 s |
| Supplemental Figure 5c | F | 1:2 CBD | 12 | 89.16 ± 12.41 s |
| Supplemental Figure 5c | F | 1:1 CBD | 12 | 52.35 ± 9.80 s |
| Supplemental Figure 5c | F | 1:0 CBD | 12 | 64.88 ± 8.87 s |
| Supplemental Figure 5c | F | OG Kush | 12 | 71.12 ± 9.49 s |

**Supplemental Table 1:** Descriptive statistics.
