# Supplementary material for: Cannabidiol and cannabis-inspired terpene blends have acute prosocial effects in the BTBR mouse model of autism spectrum disorder
Source: Front Neurosci. 2023 Jun 16;17:1185737. doi: 10.3389/fnins.2023.1185737 (PMC10311644; doi:10.3389/fnins.2023.1185737)
Supplement: Supplementary file 2 [file Data_Sheet_1.docx]

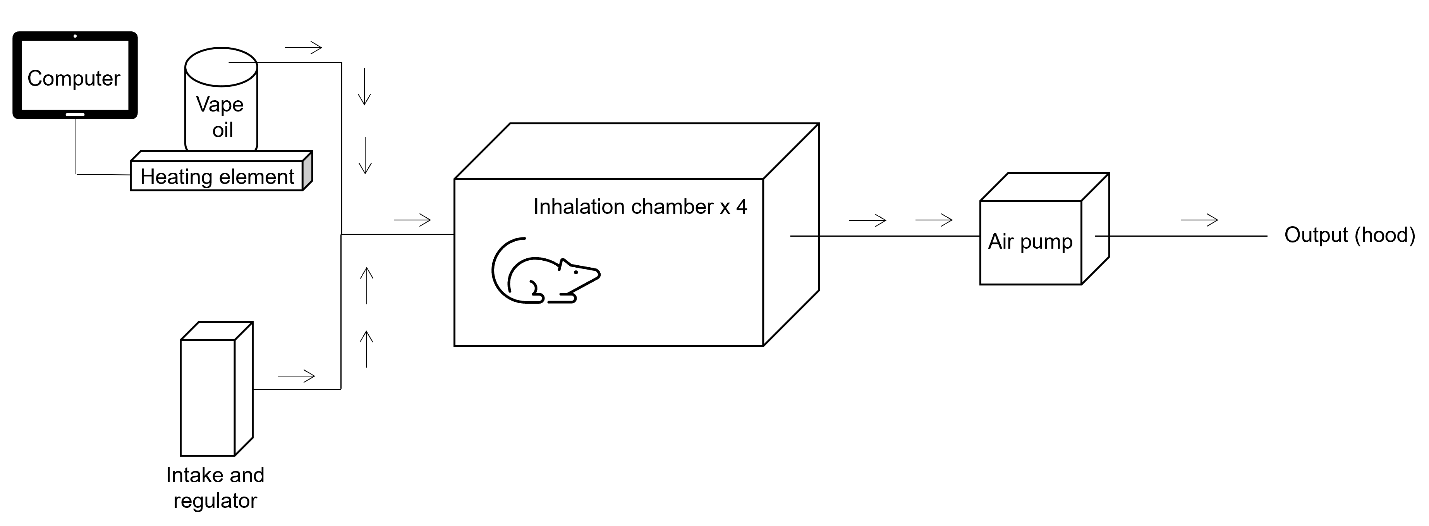
Supplemental Figure 1

| **Supplemental Figure 1:** Schematic of the passive inhalation chambers. A suction air pump draws air through an intake port and pulls vapor from a tank containing the vape oil. The air and vapor then is pulled evenly through 4 inhalation chambers and then pushed out to a fume hood. The heating element is controlled by a computer and aerosolizes the contents of the oil leading to vapor entering the chambers. |  |  |
| --- | --- | --- |
|  |  |  |
