# Supplementary material for: Cannabidiol and cannabis-inspired terpene blends have acute prosocial effects in the BTBR mouse model of autism spectrum disorder
Source: Front Neurosci. 2023 Jun 16;17:1185737. doi: 10.3389/fnins.2023.1185737 (PMC10311644; doi:10.3389/fnins.2023.1185737)
Supplement: Supplementary file 3 [file Data_Sheet_2.docx]

Supplemental Figure 2

1. **B.**

| **CBD concentration (mg/L)** | **Area**  **(counts)** |
| --- | --- |
| 100 | 1103.37 |
| 200 | 2044.46 |
| 300 | 2733.16 |
| 400 | 4072.19 |
| 500 | 5116.29 |

**C.**

| \| **Sample**  **(diluted 1:100)** \| **Area**  **(counts)** \| **CBD Concentration (mg/ml)** \| \| --- \| --- \| --- \| \| Koi \| 2141.5 \| 21.32 \| \| Blue Moon \| 2265.97 \| 22.56 \| \| Savage* \| 2436.79 \| 24.26 \| |  |  |
| --- | --- | --- | --- | --- | --- | --- | --- | --- | --- | --- | --- | --- | --- | --- |
|  |  |  |
|  |  |  |
| **Supplemental Figure 2:** CBD quantification of 3 commercial hemp oils. **A.** CBD standard curve generated with 5 CBD standard concentration. **B.** CBD standard concentrations and corresponding area counts. **C.** Each of the three commercial product samples with corresponding area counts and calculated CBD concentration. * indicates highest CBD concentration and the product that was assessed for behavioral effects throughout the manuscript. |  |  |
|  |  |  |
