# Supplementary material for: Cannabidiol and cannabis-inspired terpene blends have acute prosocial effects in the BTBR mouse model of autism spectrum disorder
Source: Front Neurosci. 2023 Jun 16;17:1185737. doi: 10.3389/fnins.2023.1185737 (PMC10311644; doi:10.3389/fnins.2023.1185737)
Supplement: Supplementary file 4 [file Data_Sheet_3.docx]

Supplemental Figure 3

1. **B.**

| **CBD concentration (mg/L)** | **Area**  **(counts)** |
| --- | --- |
| 0.01 | 11477.56 |
| 0.5 | 42988.08 |
| 1 | 90301.06 |
| 1.5 | 119281.6 |
| 2.5 | 206803.9 |

**C.**

| \| \| **Mouse Number** \| **Area (counts)** \| **Dilution Factor*** \| **Concentration (ng/ml)** \| \| --- \| --- \| --- \| --- \| \| #1 – female \| 40632.58 \| 0.2 \| 0.0841 \| \| #2 – female \| 47652.59 \| 0.2 \| 0.1020 \| \| #3 – female \| 41648.48 \| 0.2 \| 0.0867 \| \| #4 – female \| 51995.64 \| 0.2 \| 0.1130 \| \| #5 – male \| 83299.1 \| 0.2 \| 0.1927 \| \| #6 – male \| 74409.13 \| 0.2 \| 0.1701 \| \| #7 – male \| 55076.02 \| 0.2 \| 0.1209 \| \| #8 – male \| 75555.98 \| 0.2 \| 0.1730 \| \|  \|  \| \| --- \| --- \| --- \| --- \| --- \| --- \| --- \| --- \| --- \| --- \| --- \| --- \| --- \| --- \| --- \| --- \| --- \| --- \| --- \| --- \| --- \| --- \| --- \| --- \| --- \| --- \| --- \| --- \| --- \| --- \| --- \| --- \| --- \| --- \| --- \| --- \| --- \| --- \| --- \| \|  \|  \|  \| \|  \|  \|  \| \|  \|  \|  \| |  |  |
| --- | --- | --- | --- | --- | --- | --- | --- | --- | --- | --- | --- | --- | --- | --- | --- | --- | --- | --- | --- | --- | --- | --- | --- | --- | --- | --- | --- | --- | --- | --- | --- | --- | --- | --- | --- | --- | --- | --- | --- | --- | --- | --- | --- | --- | --- | --- | --- | --- | --- | --- |

**Supplemental Figure 3:** CBD quantification in plasma by LC/Q-TOF. **A.** CBD standard curve generated with 5 CBD standard concentration. **B.** CBD standard concentrations and corresponding area counts. **C.** Area counts for each subject. Note that a dilution factor of 0.2 was used in the calculation of each subject’s plasma concentration to account for the higher injection volume and addition of acetonitrile during the liquid-liquid extraction (see methods for further details).
