# Supplementary material for: Cannabidiol and cannabis-inspired terpene blends have acute prosocial effects in the BTBR mouse model of autism spectrum disorder
Source: Front Neurosci. 2023 Jun 16;17:1185737. doi: 10.3389/fnins.2023.1185737 (PMC10311644; doi:10.3389/fnins.2023.1185737)
Supplement: Supplementary file 5 [file Data_Sheet_4.docx]

Supplemental Figure 4


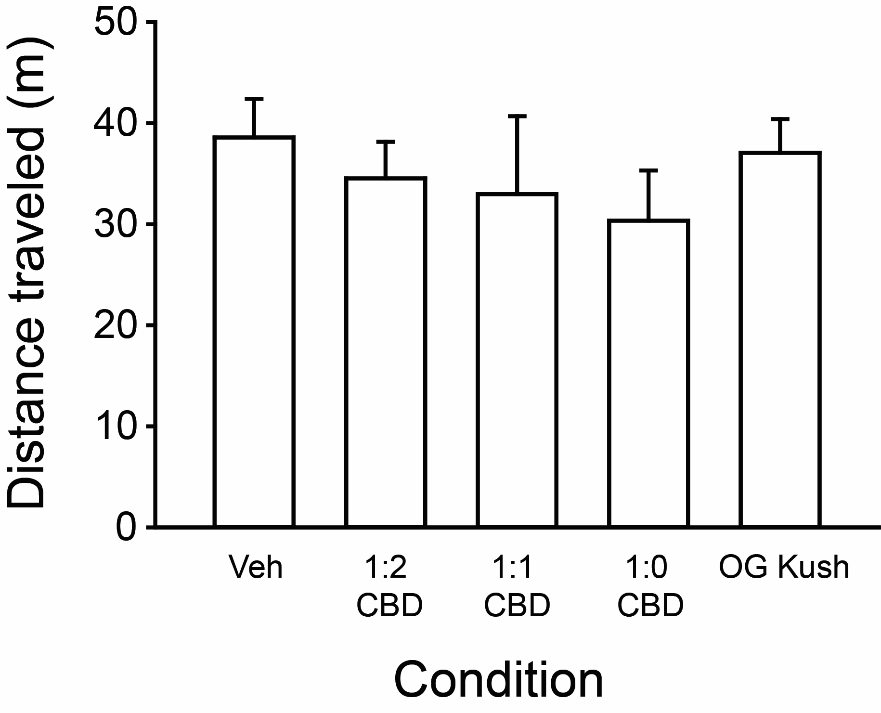


| **Supplemental Figure 4:** CBD nor OG Kush affect locomotor activity during the habituation phase of the 3 Chamber Test. This summary bar chart shows the distance traveled during the 10 minute habituation phase of the 3 Chamber Test after exposure to either vehicle (veh), one of three CBD product dilutions of CBD:vehicle (1:2, 1:1, and undiluted [1:0]), and 5% OG Kush terpenes in vehicle. There was no effect of any condition on locomotor activity, *P* = 0.78. |  |  |
| --- | --- | --- |
|  |  |  |
