# Supplementary material for: Cannabidiol and cannabis-inspired terpene blends have acute prosocial effects in the BTBR mouse model of autism spectrum disorder
Source: Front Neurosci. 2023 Jun 16;17:1185737. doi: 10.3389/fnins.2023.1185737 (PMC10311644; doi:10.3389/fnins.2023.1185737)
Supplement: Supplementary file 6 [file Data_Sheet_5.docx]

Supplemental Figure 5


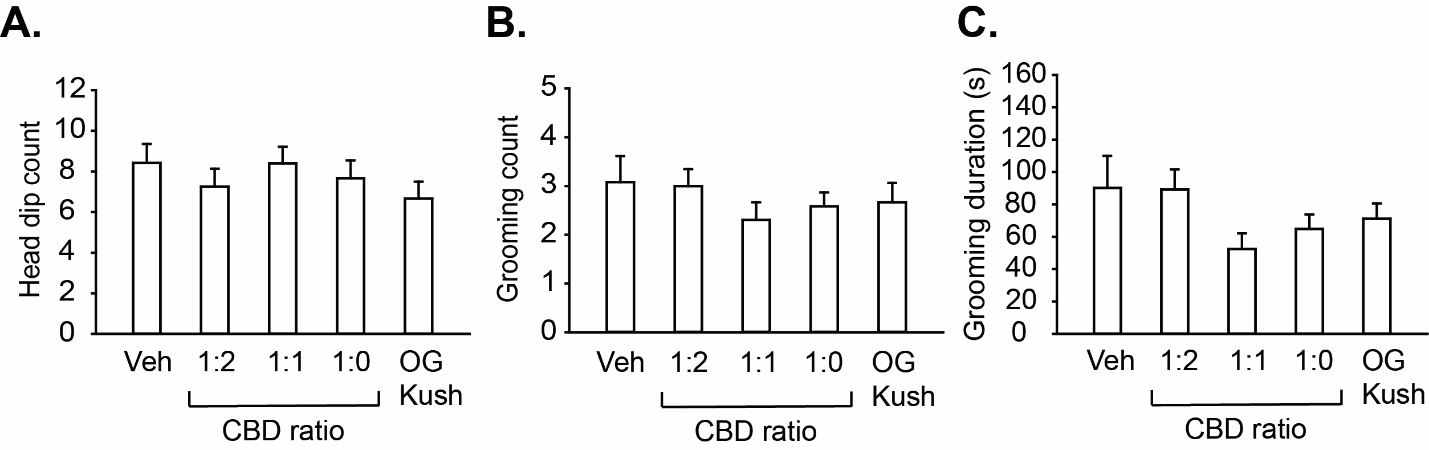


| **Supplemental Figure 5:** Lack of effects of CBD and OG Kush on repetitive behaviors on the EPM. A. Summary bar chart showing that neither CBD, at 3 different concentrations, nor 5% OG Kush affected the number of head dips. B. Summary bar chart showing that neither CBD nor OG Kush affected the frequency of grooming behavior. C. Summary bar chart showing that neither CBD nor OG Kush affected total grooming duration. All *P >* .05 by one-way between subjects ANOVA. |  |  |
| --- | --- | --- |
|  |  |  |
